# Enhancing Paediatric and Strabismus Ophthalmology Departments in Spain Through the Inclusion of Orthoptists: Insights from a Nationwide Survey

**DOI:** 10.22599/bioj.359

**Published:** 2024-12-03

**Authors:** Ana María Cruz Lasso

**Affiliations:** 1 Independent researcher, Spain

**Keywords:** Orthoptist, paediatric ophthalmology, strabismus, quality of care, professional competences, questionnaire

## Abstract

**Background and objectives:**

The absence of the orthoptic profession in Spain contrasts with its value and recognition in other countries. This study aimed to gauge Spanish ophthalmologists’ interest in including and promoting orthoptists in paediatric and strabismus departments. Secondary objectives included assessing interest in requesting orthoptists, involvement in their training, and aligning their competencies with European standards.

**Methodology:**

Participants were recruited via WhatsApp and flyers during the 30th SEEOP Congress in May 2023 and invited to review a report on orthoptists’ significance followed by completing a 12-question online survey. Profile questions were asked via multiple-choice options. Opinions on integrating orthoptists to enhance care quality, training involvement, and alignment with European standards were rated using a Likert scale. Open-ended questions captured themes, and comments.

**Results:**

Forty-two paediatric and strabismologists in Spain participated, mostly hospital-employed with advanced degrees, 90% supported integrating orthoptists into their teams, with 83% interested in having them in their workplace. Nearly 90% believed that collaboration between ophthalmologists and orthoptists would improve care quality and reduce waiting lists, and 83% supported promoting this within national medical organisations. Additionally, 90% advocated level 4 advanced European-standard training for orthoptists and 25% were neutral about participating in training orthoptists. Participants emphasised the importance of qualified orthoptists for treating eye movement abnormalities, distinguishing them from other eye healthcare professionals and advocating for collaboration rather than replacement.

**Conclusions:**

This initial survey of paediatric ophthalmologists and strabismologists in Spain highlights support for orthoptists as allied health professionals, though garnering support for their training could be challenging. These conclusions should be considered in light of methodological, sample size, and resource limitations. The survey serves as a pilot for the future, suggesting improvements to explore the feasibility of introducing orthoptists in Spain.

## Background and Objectives

Abnormalities of binocular vision and visual development are managed by a team of specialists comprising paediatric ophthalmologists, strabismologists, orthoptists, optometrists and vision scientists who ensure high-quality care for paediatric and strabismus populations (AAO, 2022).

The field of paediatric ophthalmology and strabismus encompasses a broad spectrum of conditions that impact both anterior and posterior segments of the eye visual and efferent pathways. These include amblyopia, cataract, nystagmus, retinopathy of prematurity, and strabismus (Lueder *et al*., 2023; Weinert and Heidary, 2021). Professionals in this field are required to possess specialised skills, patience, and allocate substantial time for eye examinations to ensure the delivery of the highest quality of care (Mezad-Koursh *et al*., 2022; Miller, 2015). However, current evidence highlights a shortage of practicing paediatric ophthalmologists, severely limiting access to comprehensive eye care for the paediatric population, which compromises the quality of care (Bernstein and Nelson, 2020; Dotan, Karr, and Levin, 2017; Lee *et al*., 2022).

In Spain ophthalmology services are the second most sought-after specialty after traumatology (SISLE-SNS, 2023). Notably, it ranks among the specialties receiving the highest number of referrals from primary care (Figueras-Roca *et al*., 2019). This trend is expected to intensify due to the global increase in the elderly population, leading to a higher prevalence of age-related eye problems worldwide (Claxton, 2022; Garcia, Garcia, and Markides, 2019; Oohira, 2022). Consequently, ophthalmological services are grappling with mounting capacity pressure and burnout, primarily attributed to excessive workloads (Cheung *et al*., 2021; Garrido-Hermosilla *et al*., 2021).

After gaining a bachelor’s degree in medicine, ophthalmologists in Spain undergo a minimum of four years of training in ophthalmology (SAS, 2009). Meaning studying a minimum of 11 years (OftalmoSEO, 2024). There are currently 3335 registered ophthalmologists (SNS, 2024) for 48.69 million population or 1:14600 population.

To uphold the recommended care quality standards set by the World Health Organization (WHO, 2022a), innovative measures are essential. A crucial aspect involves strengthening multidisciplinary collaboration and nurturing connections among healthcare professionals dedicated to advancing eye services (Lee *et al*., 2023; Tzoumas, 2020). In the context of paediatric and strabismus ophthalmology departments, orthoptists can assume a crucial role. Their significance is detailed as follows.

Orthoptists are allied health professionals with highly specialised knowledge and skills focussed on investigating, diagnosing, and managing eye movement abnormalities in both children and adults (AAPOS, 2022; OCE, 2022a; BIOS, 2023). Orthoptists go beyond their traditional roles, providing expertise to low vision patients and those with visual impairments from accidents or strokes (Jansen *et al*., 2020). They also play a significant role in providing orthoptic care for individuals with physical and mental disabilities, as well as adults with learning disabilities (BOD, 2022; Diplock and Mehta, 2021; Fanning and O’Connor, 2021). This work is both demanding and rewarding (OCE, 2022a). It’s crucial to underscore that the orthoptic profession is distinct from behavioural vision therapy providers (Rucker and Phillips, 2018; Shainberg, 2010).

Orthoptists form close collaborations with ophthalmologists in units specialising in strabismus, ocular motility, neuro-ophthalmology, and paediatric ophthalmology (Horwood, 2016; IOA, 2022a). Moreover, their skills are utilised by physicians, facial-maxillary surgeons, and other healthcare professionals to assist in diagnosing complex eye movement disorders and visual field loss, providing valuable support to patients experiencing symptoms including double vision (Gregory, 2013; OCE, 2022a).

Orthoptists work in various settings, such as tertiary eye centres, university eye clinics, general hospitals, and neurological clinics, as well as early intervention centres, rehabilitation facilities, and institutions for the visually impaired and blind. Furthermore, orthoptists actively participate in vision-related research, contribute to international projects, and engage in education programmes. The impact of their involvement is demonstrated by high-quality research published in strabismus and orthoptic journals (Kraft, 2013). The widespread network of orthoptic centres highlights the effective alignment between undergraduate teaching and the expectations of newly graduated professionals. This alignment is further demonstrated in the close agreement between orthoptists and medical practitioners during comprehensive eye examinations, affirming the effectiveness of their education, training, and skills (Horwood, 2018; Scheetz *et al*., 2016; Waters *et al*., 2021).

The significance of the orthoptic profession spans from its inception in 1930s (Brown, 2020; Flynn, 1991) to the present day. This is highlighted by growing recognition from various international medical societies and associations (AAPOS, 2022; IOA, 2022b; IPOSC, 2022; ISA, 2022) in Europe and worldwide (IOA, 2022c; OCE, 2022b). Orthoptics is a distinct profession, enjoying state registration and ‘protected title’ status in numerous countries, such as the UK, Netherlands, USA, Australia, and others. Furthermore, the Orthoptistes de la Communauté Europeanne (OCE) have actively worked towards establishing a common standard of Orthoptic Education in Europe (OCE, 2024), emphasising the profession’s continuing relevance and commitment to excellence.

Despite the widespread recognition and well-established presence of orthoptists in numerous countries, and the urgent need to strengthen multidisciplinary collaboration among health professionals for enhanced quality of care, it is striking that the orthoptic profession is still not recognised in Spain.

Considering its rich historical background and the varied clinical and social benefits it offers, integrating the orthoptic profession into paediatric and strabismic ophthalmology services in Spain seems desirable.

As a result, this study aimed to survey Spanish strabismologists and paediatric ophthalmologists to find the level of their interest in the inclusion and promotion of orthoptists in paediatric and strabismus ophthalmology departments in Spain. Secondary aims included assessing specific aspects such as the participants’ interest in requesting orthoptists in their workplace, their involvement in training and their expectation for aligning orthoptists’ competencies with the level 4 advanced orthoptics (Table A.1) of the European Competences Profile. Additionally, the study seeks to know ophthalmologists’ expectations regarding the orthoptists’ role in investigating, diagnosing, and managing eye movement abnormalities. This is the first survey of its kind and aims to open the debate on the need for orthoptists for the population of Spain.

## Methodology

### Identification and recruitment

Initially, a Spanish specialist in child and adult strabismus and paediatric ophthalmology was contacted to provide a review of the study’s objectives. This ophthalmologist generously distributed the questionnaire to colleagues within their professional network for feedback. Participants were invited using emails and social media platforms like WhatsApp and LinkedIn (Gaur *et al*., 2020). Subsequently, additional recruitment was conducted during the 30th Congress of the Spanish Society of Strabology and Paediatric Ophthalmology (SEEOP) by personal invitations.

Survey data was collected anonymously through the online questionnaire application ‘forms.app,’ providing participants with the sole option to respond digitally. This method ensures the privacy and confidentiality of respondents while efficiently gathering their input.

Prior to the survey, participants were encouraged to review a report that gathers relevant information about orthoptics. This document (Cruz Lasso, 2022) addressed issues such as defining orthoptics, the historical and current background, why this profession is recognised in other countries worldwide, their significance in improving the quality of care in paediatric and strabismus ophthalmology departments and their competencies, agreed upon by the European community, among others (see annex).

The target ophthalmologists were encouraged to review the article posted at the link: https://orthopticspain.blogspot.com/.

Ethical approval for the survey’s content and accessibility was obtained from forms.app, an EU-based server (forms.app, 2023). The collected data undergoes encryption and secure storage by forms.app EU Services, ensuring strict adherence to the European Union General Data Protection Regulation (GDPR). Participants granted consent for their responses to be included in reports or manuscripts at the time of survey completion.

**Figure 1:**
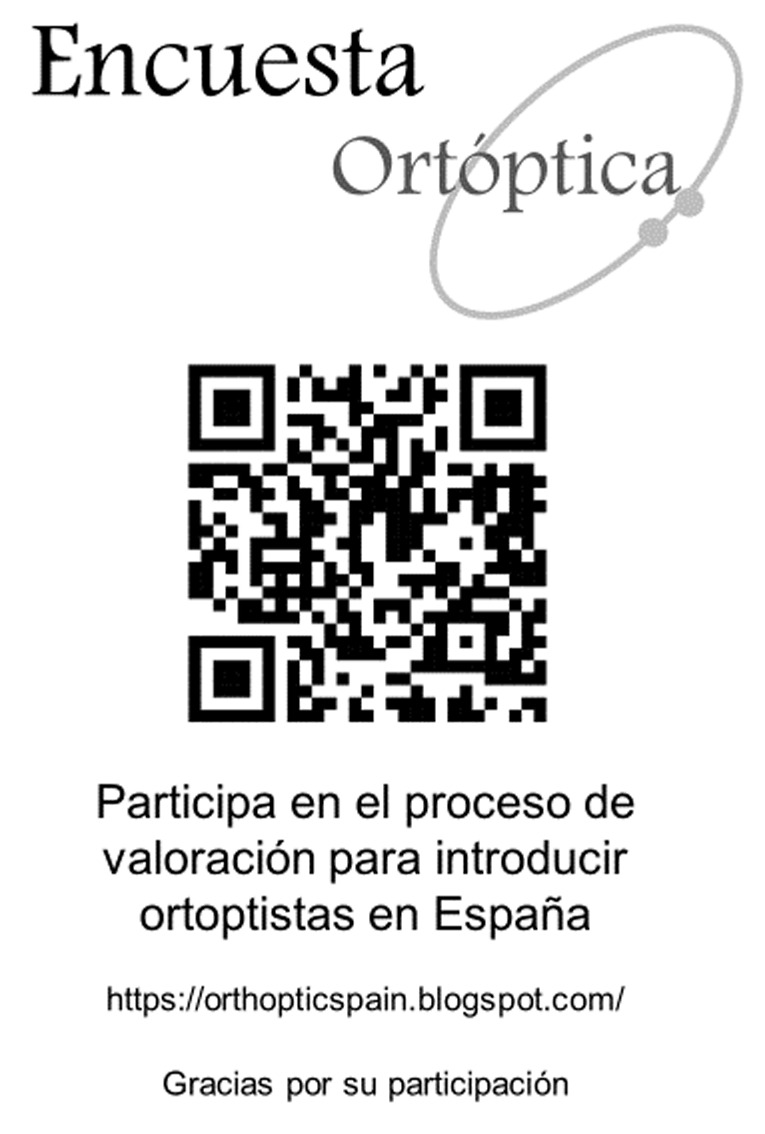
Flyer distributed at the 30th Congress of the Spanish Society of Strabology and Paediatric Ophthalmology (SEEOP).

The report and the survey were distributed in two phases: firstly, over a one-month period through the WhatsApp application among paediatric ophthalmologists andstrabismologists. Subsequently, during the 30th Congress of the Spanish Society of Strabology and Paediatric Ophthalmology (SEEOP) held in May 2023, flyers with a QR code (see Figure 1) were provided to facilitate access to a blog (Orthoptic Spain, 2023) created to provide information about the study’s objectives and links to the report (Cruz Lasso, 2022) and the survey. This multiple distribution helped ensure that the report and the survey were accessible to a large number of professionals, both digitally through WhatsApp and physically through conference flyers.

The survey contains 12 short questions that cover four main categories (see Table 1):

- Block 1: Q1–4 Participant profile.
- Block 2: Q5–7 Interest in the inclusion and promotion of the orthoptics profession in Spain.
- Block 3: Q8–10 Interest in requesting orthoptists, level of orthoptist training and level of involvement in their training.
- Block 4: Q11–12 Open-ended questions: what is expected from an orthoptist and suggestions, proposals, opinions, and comments.

**Table 1:** Survey after reading the article: ‘Enhancing Paediatric and Strabismus Ophthalmology Departments in Spain Through the Inclusion of Orthoptists: Insights from a Nationwide Survey.’


**Questionnaire after reading the article:** ‘Enhancing Paediatric and Strabismus Ophthalmology Departments in Spain Through the Inclusion of Orthoptists: Insights from a Nationwide Survey’

**1. Name of the city where you work**.

**2. Job position (you can select more than one option):** Hospital Clinic University

**3. Years of professional experience in strabismus and paediatric ophthalmology clinics.** Less than 3 years 3 to 6 years 6 to 10 years More than 10 years

**4. Academic qualifications:** Bachelor’s Degree Master’s Degree Doctorate

**5. To what extent do you think the inclusion of the orthoptics profession in strabismus, ocular motility, neuro-ophthalmology, and paediatric ophthalmology units in Spain is necessary?** Not necessary Slightly necessary Neutral Necessary Very necessary

**6. To what extent do you believe that the collaboration between ophthalmologists and orthoptists would improve the quality of care and reduce waiting lists?** Does not improve Slightly improves Neutral Improves Significantly improves

**7. To what extent do you believe it is important for Spanish ophthalmological societies and associations to align with the demands for the promotion of orthoptists, as international medical organisations do?** Not important Slightly important Neutral Important Very important

**8. To what extent do you believe it is necessary for orthoptists in Spain to meet the advanced level of training, as established in the EDORTH project (see annex)?** Not necessary Slightly necessary Neutral Necessary Very necessary

**9. To what extent would you be interested in requesting orthoptists in your workplace?** Not interested Slightly interested Neutral Interested Very interested

**10. Would you be interested in getting involved in the training of orthoptists if it were required?** Not interested Slightly interested Neutral Interested Very interested

**11. What would you expect from an orthoptist if you were to work with them?**

**12. Below, you can provide your opinion, comments, proposals, or suggestions**.

Thank you for your cooperation.

All results that may be published will be completely anonymous, which means that no participant will be identifiable.


Translated from Spanish using Google translate. See Spanish version in Annex.

It is important to note that the survey was provided in Spanish (see annex).

The objectives of each block are different; therefore, different types of responses have been designed:

- In the first block, information was collected to understand the participants’ profiles: questions were asked about the city and workplace, years of professional experience, and academic background. For analysis, single-choice responses were used for the city of work, while multiple-choice responses were used for the rest.
- In the second block, the aim is to determine the extent to which participants believe it is necessary to include orthoptists in paediatric ophthalmology and strabismus clinics, whether it would improve the quality of care, reduce waiting lists, and whether their promotion within national medical organisations would be beneficial. For assessment, responses have been designed using a Likert scale with five response options.
- In the third block, questions are posed regarding the involvement of the medical community: whether participants would personally request orthoptists in their workplace, whether they would be involved in their training, and finally, whether orthoptists should receive the advanced level of training according to European competencies (OCE, 2023). For assessment, responses have also been designed using a Likert scale with five response options.
- In the fourth and final block, the aim is to understand, on one hand, the expectations regarding orthoptists, and on the other hand, to gather information about proposals, suggestions, and comments from the respondents. To gather all this additional information, open-ended questions have been formulated.

## Results

### Quantity and distribution of responses

A total of 42 responses have been obtained from paediatric ophthalmologists and strabismologists.

**Figure 2:**
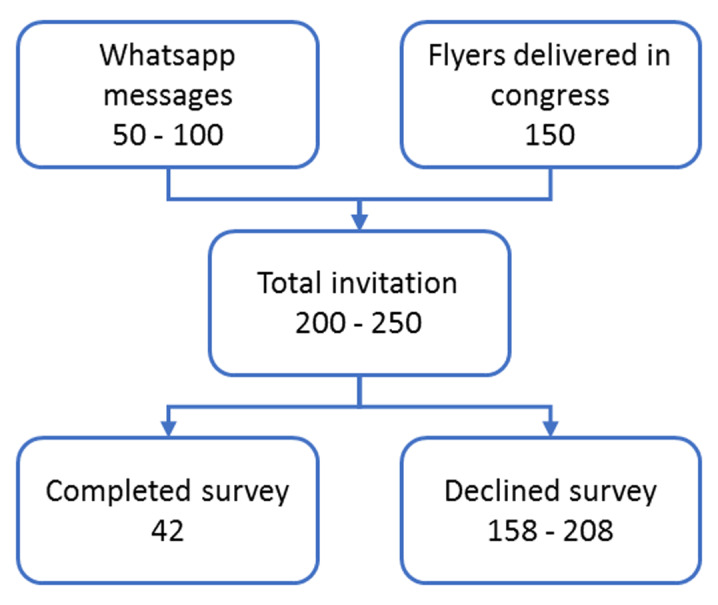
Survey response flow chart.

If approximately 50 to 100 specialists were approached through WhatsApp groups and around 150 flyers were distributed during the Congress, it is estimated that approximately 200 specialists received the survey – about 6% of the total Spanish ophthalmologists. Figure 2 illustrates these results.

### Block 1: Participant profile

#### Q1. Distribution

**Figure 3:**
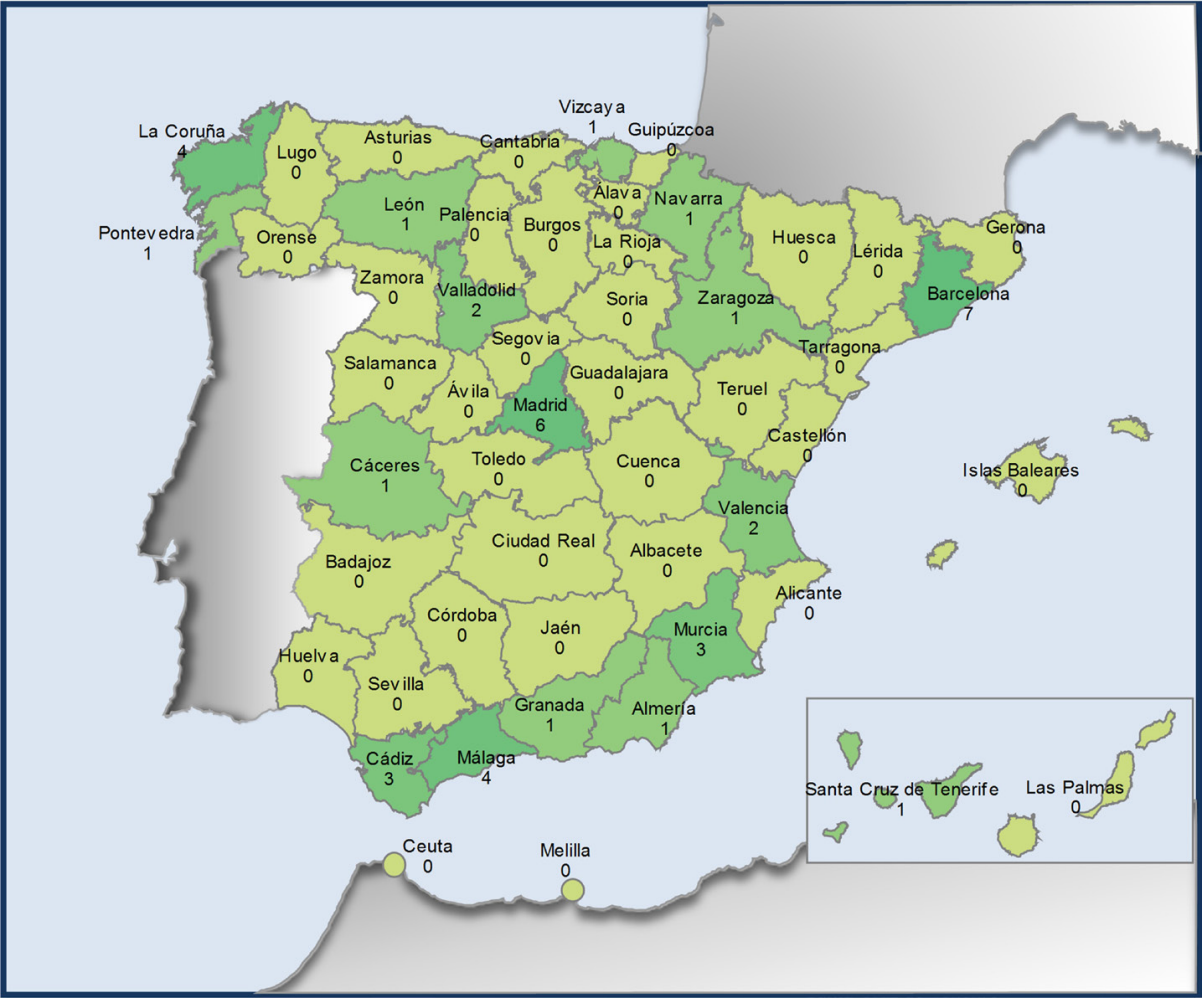
Map of the geographical distribution of responses to the survey within Spanish territory.

The workplaces are primarily located within the Spanish territory, from 17 Spanish provinces. The summary of this distribution is displayed in Figure 3. The map was generated utilising the survey data through the application: https://analisisydecision.es/nuevo-y-muy-mejorado-mapa-de-espana-por-provincias-con-excel/. While there is a general geographical distribution of respondents’ workplaces, some areas are not represented.

The respondents mostly used their mobile devices to complete the questionnaire (approximately 71%), while the remaining used desktop computers (about 29%). The average time to complete the survey was 3 minutes and 37 seconds.

#### Q2. Job position

In this question, participants were allowed to select more than one workplace option, from hospital, clinic, and university. The majority are clinicians exclusively dedicated to hospital work, while the rest work in clinics. Additionally, 6 out of the 42 participants combine their work in clinics and hospitals, and the 3 clinicians who indicate working at a university, are also affiliated with either a clinic or a hospital.

#### Q3. Years of professional experience

**Figure 4:**
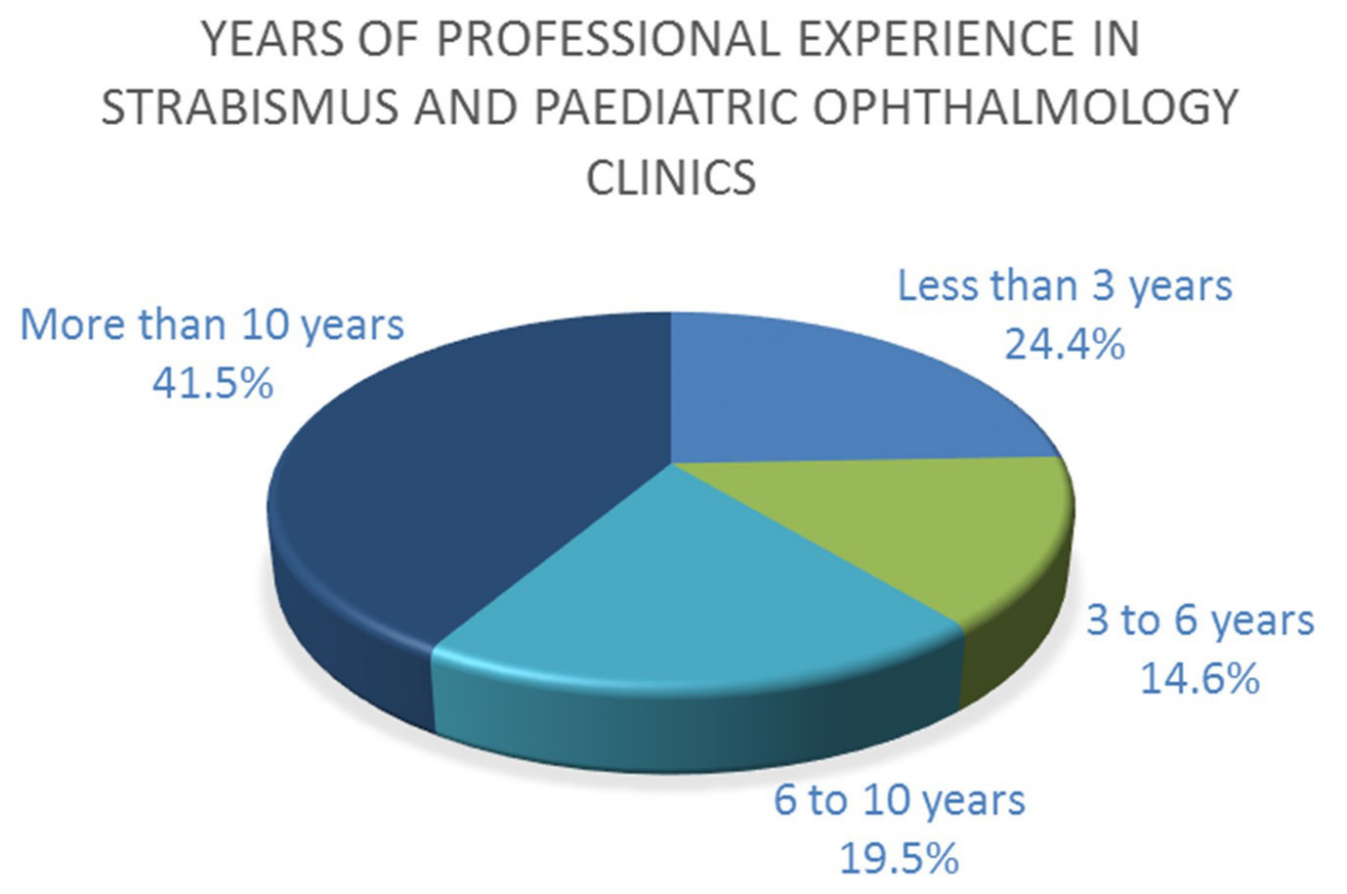
Years of professional experience in strabismus and paediatric ophthalmology clinics of the respondents.

Answers show that 41.5% had more than ten years of experience, 24.4% had less than three years, 19.5% had between six to ten years, and 14.6% had between three to six years of experience. Figure 4 summarises this information.

#### Q4. Academic qualifications

**Figure 5:**
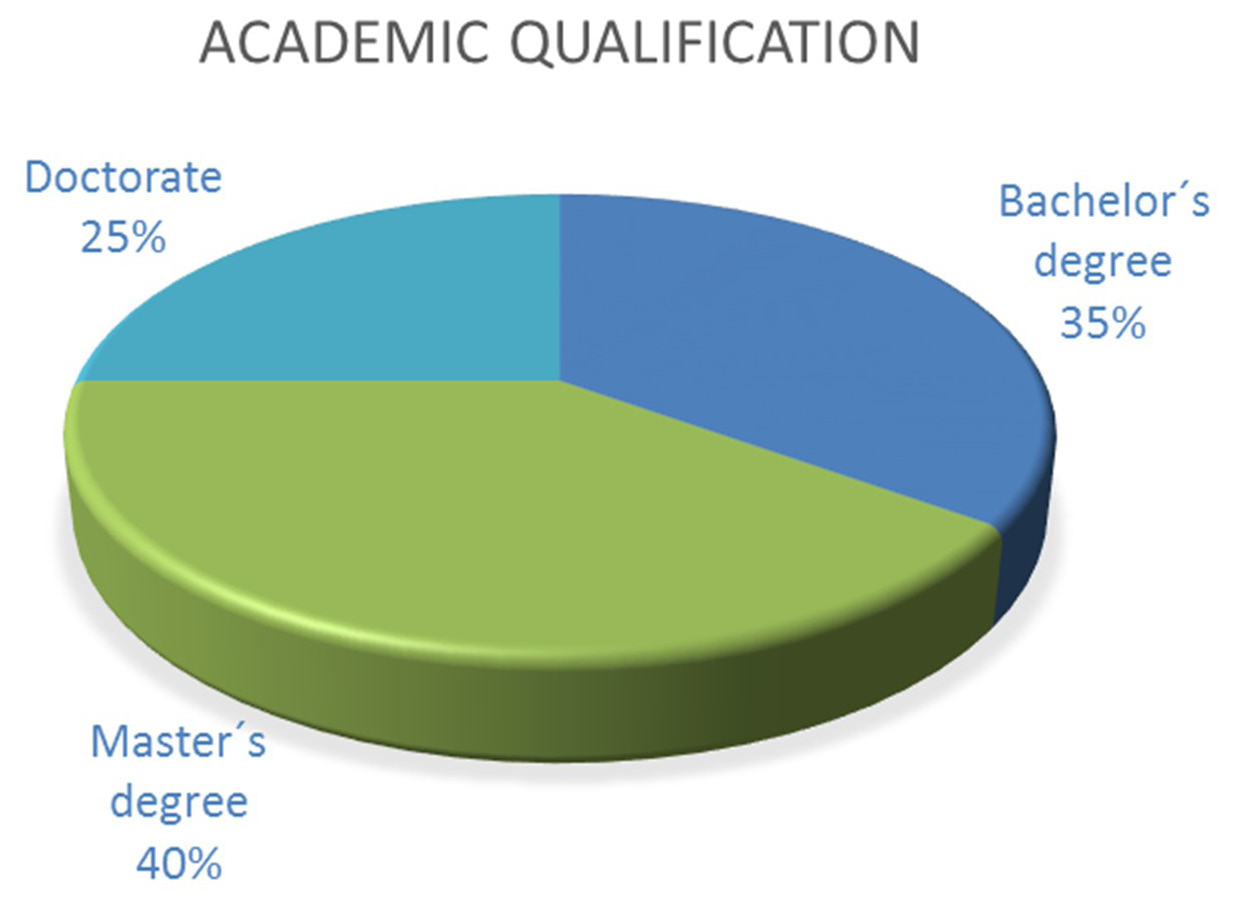
Academic qualifications of the respondents.

The responses indicate that 35% have only a bachelor’s degree, 40% have both a bachelor’s and a master’s degree, and 25% have a bachelor’s degree and a PhD. Figure 5 provides this information graphically.

### Block 2: Interest in the inclusion and promotion of the orthoptics profession in Spain

**Figure 6:**
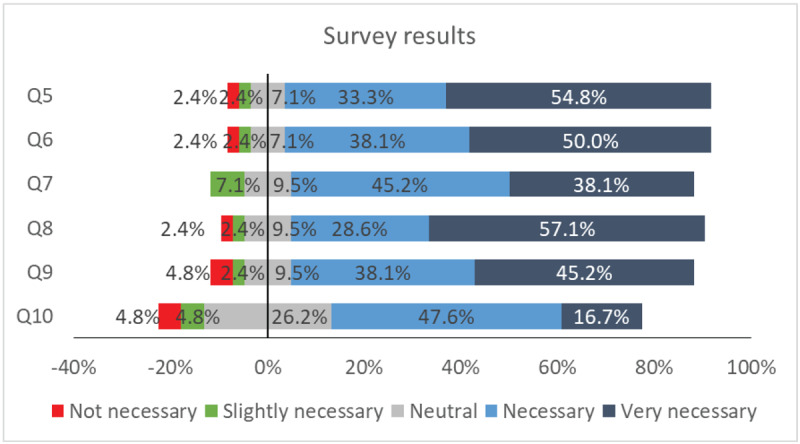
Percentage of the answers from question 5 to 10.

The results of the questions from the 5 to 10 (block 2 and 3) are represented in Figure 6. The total answers of the block 2 are shown as supplementary data located in the annex (Table A.6).

#### Q5. Need for the inclusion of the orthoptics profession in strabismus, ocular motility, neuro-ophthalmology, and paediatric ophthalmology units

Among respondents, 54.8% consider it very necessary, 33.3% consider it necessary, 7.1% remain neutral, 2.4% consider it slightly necessary, and another 2.4% do not consider it necessary.

#### Q6. Improvement of the quality of care and reduction of waiting lists through ophthalmologist-orthoptist collaboration

Approximately 50.0% of professionals believe it would lead to a significant improvement, while 38.1% are of the opinion that it would result in improvement. About 7.1% of the respondents maintain a neutral stance, whereas 2.4% believe it would result in slight improvement, and an additional 2.4% assert that it would not improve at all.

#### Q7. Degree of importance in promoting the role of the orthoptist within Spanish ophthalmological societies and associations

Of the respondents, 45.2% deem it as an important factor, while 38.1% regard it as very important. A smaller portion, 9.5%, maintain a neutral standpoint, and the remaining 7.1% perceive it as slightly important.

### Block 3: Interest in requesting orthoptists, level of orthoptist training and level of involvement in their training

#### Q8. Advanced level of training for orthoptists, according to European competencies

A majority of respondents, 57.1%, believe it is essential for the competencies of orthoptists to align with the advanced level 4 as defined in the EDORTH project. While the 28.6% express a degree of necessity for this alignment, 9.5% remain neutral, 2.4% consider it slightly necessary and another 2.4% do not regard it as necessary.

#### Q9. Interest in demanding orthoptists in their own workplace

Among the respondents, 45.2% express a high level of interest in advocating for the inclusion of orthoptists in their workplace, 38.1% are interested in this proposition, while 9.5% remain neutral, 2.4% shows a slight interest and 4.8% indicate no interest in such an inclusion.

#### Q10. Interest in getting involved in providing training for orthoptists, if required

Of the participants, 47.6% are inclined to offer training opportunities for orthoptists. Among them, 16.7% display a significant interest, while 26.2% maintain a neutral stance on the matter. A smaller fraction, 4.8%, exhibits a slight interest, and an equal percentage, 4.8%, indicates no interest.

Table 2 shows the statistical analysis of the questions from Q5 to Q10 according to the Likert scale from 0 to 4, considering the limitations inherent in the survey, see discussion section. A score of 0 signifies ‘not important’ or ‘not necessary’, while 4 denotes ‘very important’ or ‘very necessary’. The average and median indicate that the answers tend to fall within the range of agreement with the questions, with values of 3 and 4. The standard deviation, being less than 1, suggests that the answers are not very dispersed in relation to the mean, indicating a general agreement in the responses.

**Table 2:** Statistical analysis of the Likert scale questions from Q5 to Q10.


	AVERAGE	MEDIAN	STD. DEVIATION

**Q5**	3.36	4	0.91

**Q6**	3.31	3.5	0.90

**Q7**	3.14	3	0.87

**Q8**	3.36	4	0.93

**Q9**	3.17	3	1.03

**Q10**	2.67	3	0.98


### Relationship between years of experience and Q5

**Figure 7:**
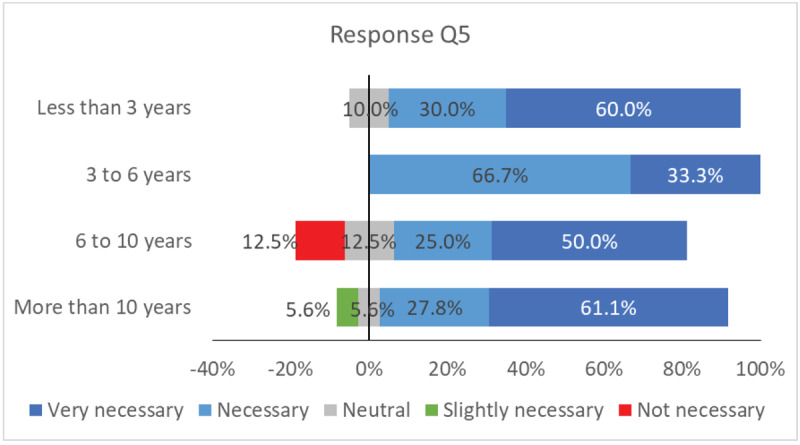
Relationship between the years of experience and the type of answer.

Figure 7 shows the relationship between years of experience and opinions on the inclusion of orthoptists in strabismus, ocular motility, neuro-ophthalmology, and paediatric ophthalmology units in Spain (Q5).

The neutral position is represented by the black vertical line. Most responses from all ranges of years of experience lean towards positive answers, with only a small percentage against. There is a small percentage of responses moving towards the negative side, when the years of experience increase.

### Consistency of responses between Q5 and Q6–10 of the survey

**Figure 8:**
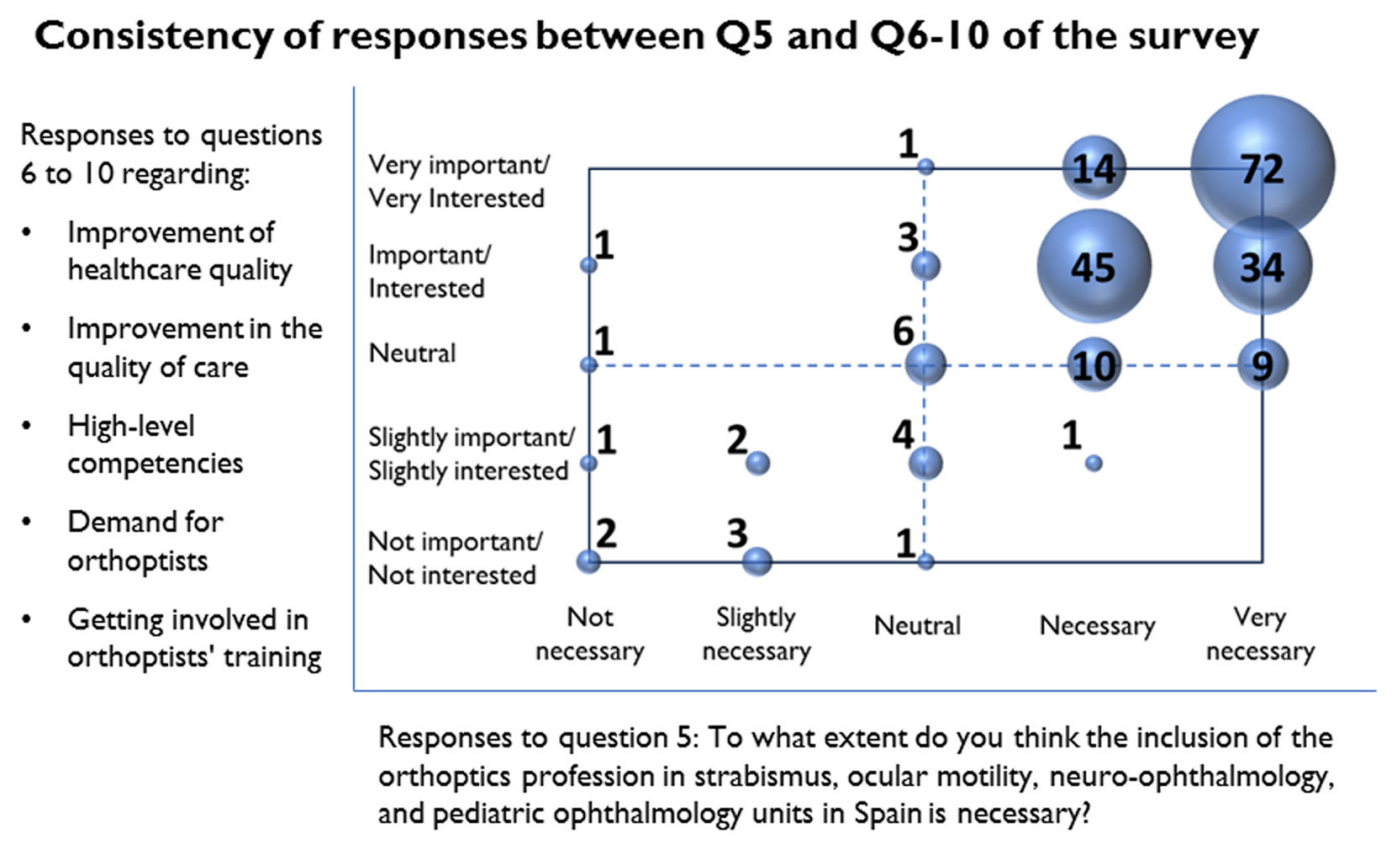
Consistency of responses between Q5 and Q6–10 of the questionnaire.

To assess the consistency of the results, responses from blocks 2 and 3 of the questionnaire were compared. Figure 8 illustrates the summation of response pairs. The Likert scale with the five responses to question 5 is represented on the horizontal axis, while the responses to questions 6, 7, 8, 9, and 10 are ordered on the vertical axis, also following the Likert scale. Each respondent contributes five pairs of responses to the figure, resulting from the combination of the response to question 5 with each of the responses provided for questions 6 to 10. When the pairs coincide at a point, the responses are summed.

The responses obtained exhibit a high level of consistency. This means that respondents who believe that the inclusion of orthoptists in Spain is necessary also indicate that they are interested in or consider important the issues raised in the questions from both block 2 and block 3. Conversely, if they are not in favour of inclusion, they typically respond that they have no interest or do not consider important what is proposed in either block.

### Block 4: Open-ended questions

Two text boxes were provided for participants to answer questions 11 and 12. Thematic analysis was then used to explore their responses regarding their expectations of orthoptists and their general opinions, comments, proposals, and suggestions.

#### Q11. What is expected from an orthoptist?

Only 24 out of 42 respondents answered this first open-ended question; 50% indicated having more than 10 years of experience, 21% between 6 to 10 years, 17% had 3 to 6 years, and 12% had less than 3 years of experience.

##### Summary of themes

Role expectations

The roles orthoptists will play within the healthcare system seem to be important. Having a qualified professional with expertise in strabismus would enhance efficiency and time management for specialists while providing much-needed patient support. Key tasks include high-level assessment of binocular vision and ocular motility pathology, evaluation and follow-up for children with eye patches, prism patients, and visual re-education needs. Responsibilities also involve measuring versions and ductions, grading prism deviations, and collaborating in vision therapy, refraction, oculomotor control studies, and low vision care. Quotes were translated from the Spanish using Google Translate.

Being able to have a qualified professional with expertise in strabismus to help me perform my work more efficiently and optimise my time. At the same time, patients would find support and solutions in a field that is currently almost deserted.

High-level assessment of binocular vision and ocular motility pathology.

Evaluation and follow-up of children with eye patches, patients with prisms, assessment of the need for visual re-education.

Measurement of versions, ductions. Graduation of prism deviations. Collaboration in vision therapy.

Collaborate in refraction, in the study of oculomotor control, in performing tests, and in vision therapy and low vision, if needed.

##### Collaboration and teamwork

Collaboration and teamwork are crucial for orthoptists, emphasising their close work with ophthalmologists and other healthcare professionals. Orthoptists are expected to integrate seamlessly into existing healthcare teams, complementing patient care and working under ophthalmologists’ supervision.

Comprehensive and collaborative examination and treatment of patients.

Collaboration and not autonomous/independent work of the orthoptist. Always under the supervision of the ophthalmologist.

##### Attributes

Professionalism, patience, and initiative are also highlighted as important for delivering high-quality, patient-centred care and positively contributing to the healthcare team and environment.

Professionalism, patience, initiative.

##### Training

Aligning orthoptist training with international standards is emphasised to ensure they are well-prepared. Proper training and qualifications are essential for effectively supporting patient care and meeting healthcare system demands.

I expect from them what is expected according to the international training program. They are crucial when making surgical.

#### Q12. Opinion, comments, proposals, or suggestions

##### Clarification of roles and competencies

Respondents express the need to distinguish between different healthcare professions. They suggest that orthoptists should not replace other healthcare professionals, but instead collaborate with them to optimise patient care. They highlight concerns about negative perceptions regarding non-evidence-based visual therapies, clarifying that orthoptists offer distinct services. Additionally, they oppose granting orthoptists competencies for medication administration.

They wouldn’t need to replace the position of an ophthalmologist or a nurse; instead, more space in hospitals would be needed, and everyone should know their role.

I believe that ophthalmologists are somewhat hesitant due to the negative impression we have of the ‘therapies’ performed by optometrists. That’s why I think it’s essential to emphasise that they are entirely different.

I believe it’s not appropriate to grant competencies for the administration of medications.

##### Support and necessity

Participants highlight the current deficiency of orthoptists in the Spanish National Health System. Collaboration between ophthalmologists and orthoptists is seen as beneficial for treating certain conditions. The presence of both orthoptists and optometrists in ophthalmology services is demanded. Specifically, orthoptists are considered essential for strabismus consultations, emphasising the valuable contributions these professionals can make to patient care.

I have always considered it a significant mistake that there is no orthoptist figure in Spain, which exists in other countries.

Collaboration between ophthalmologists and orthoptists for the treatment of certain diseases would be advisable.

I believe that orthoptists should be incorporated into ophthalmology services nationwide, and optometrists as well.

The orthoptist is indeed essential in a strabismus consultation. I fully support the inclusion of this profession as a support to the strabismologist.

## Discussion

Surveys remain the foundation of social science research and can be employed in almost any discipline, including medical research (Story and Tait, 2019). Moreover, surveys could be the primary methodology to explore a hypothesis until it evolves into a more sophisticated and validated idea, at which point it can be probed further in a systematic and structured manner using other research methods (Gaur *et al*., 2020). For this reason, a survey-based study was chosen as the research methodology to gauge the opinions of paediatric ophthalmologists and strabismologists regarding their interest in promoting the orthoptics profession before conducting a more in-depth and extensive investigation.

The lack of professional regulation for orthoptists prompted a survey to gather insights from Spanish strabismologists and paediatric ophthalmologists on integrating and supporting orthoptists. Since it is the first survey of its kind, the feedback obtained can guide the development of more focussed questions for future studies.

### Pre-survey informative document

Before responding to the questionnaire participants were encouraged to review a report by the author that compiles relevant information about orthoptists (Cruz Lasso, 2022). The document addresses issues including the role of an orthoptist, background, global recognition, significance in paediatric and strabismus ophthalmology, and competencies agreed upon by the European community. This aimed to inform respondents prior to assessing their support for the orthoptic profession.

Providing information before a survey is similar to feedback forms given at medical conferences (Gaur *et al*., 2020). This approach was necessary due to the lack of knowledge about orthoptics which could have led to respondents lacking sufficient background knowledge or with mistaken ideas of what the Orthoptist role entails (Barabas and Jerit, 2010). Informative documents help correct misconceptions, as the underinformed are more likely to update their beliefs than the misinformed (Li and Wagner, 2020). It was essential to clarify the role of orthoptists as distinct from behavioural vision-therapy providers, as the roles can be confused (Barrett, 2009; Rucker and Phillips, 2018; SEDOP, 2019; SEOptometria, 2019; Shainberg, 2010; Wang and Kuwera, 2022).

However, a common critique is the potential for bias when participants deduce the experiment’s purpose and align their responses with the researcher’s hypothesis, often leading to positive results (Mummolo and Peterson, 2019). Additionally, providing information can increase knowledge and alter previously held views and attitudes. Thus, methodological caution is needed when extrapolating from survey experiments (Barabas and Jerit, 2010), and achieving a higher response rate helps reduce the likelihood of non-responder bias (Story and Tait, 2019).

### Survey methodology

Although most questions in this study were closed-ended, two open-ended questions were included to capture themes not covered by structured questions (Gaur *et al*., 2020). Open-ended questions allow respondents to answer in their own words, reflecting personal experiences and beliefs, and are less influenced by investigator expectations (Story and Tait, 2019). They provide detailed insights and help develop new response options for future closed-ended questions. However, open-ended responses can be long, difficult to transcribe, and classify, potentially leading to incomplete answers due to response fatigue (Story and Tait, 2019; Taherdoost, 2019).

The Likert scale was chosen for its widespread use and reliability in social science research, offering simplicity in construction, standardised responses, and ease of analysis. It is also user-friendly and quick for respondents (Gaur *et al*., 2020; Story and Tait, 2019; Taherdoost, 2019; Younas and Porr, 2018). Typically, it ranges from ‘strongly agree’ to ‘strongly disagree’, with five-point scales being common. However, seven-point scales are more accurate, but a five-point scale was used here to reduce confusion and increase response rates (Taherdoost, 2019).

Despite its strengths, the Likert scale has weaknesses, such as challenges in demonstrating validity and reproducibility (Taherdoost, 2019). Additionally, this study’s scale was imbalanced, favouring neutral and positive responses, which could bias results. For example, it used options like ‘Not Necessary’, ‘Slightly Necessary’, ‘Neutral’, ‘Necessary’, and ‘Very Necessary’ instead of the classic ‘Strongly Disagree’ to ‘Strongly Agree’. Recognising and addressing these issues can help improve future survey designs.

### Sample size

Based on data from the Spanish Ministry of Health (SMH, 2024), there are 3,335 ophthalmologists in Spain (SNS, 2024). With a sample of 42 responses, this represents 1.26% of the ophthalmologist population. The survey targeted paediatric ophthalmologists and strabismologists. However, the total number of individuals in this target population is unknown, making it difficult to determine the precise representativeness of the sample.

While the 42 responses could be considered a limited sample size, studies indicate that size is not the most critical factor in measuring the quality of a study. A low response rate does not necessarily mean low quality, and a high response rate does not necessarily mean high quality and appropriateness. Instead, the goal is not always to secure the highest response rate possible, but to obtain the highest quality responses that result in a sample appropriate for the study’s goals (Holtom *et al*., 2022). Additionally, small pilot studies, typically involving 15–30 participants, are effective for assessing preliminary psychometric properties and feasibility for larger studies (Coleman and Briggs, 2002; Younas and Porr, 2018). This survey aligns with these principles.

However, saying that a sample is representative is meaningless unless researchers specify what population it represents or how its results are being applied (Rudolph *et al*., 2023). In this study, the estimate obtained in the sample cannot be generalised to the target population. Furthermore, the most crucial aspect of sample size in surveys is the number of respondents providing the necessary data, not merely the number of invitations sent or questionnaires returned (Walters, 2021). This emphasises the importance of data quality over quantity. This study shows that it follows these principles.

In this study, although the small sample size limits representation, the survey is useful for identifying trends within paediatric ophthalmologists and strabismologists. Therefore, the data should be contextualised within this subgroup and the survey’s intent. Limited resources and the single-researcher approach influenced the sampling. These constraints will be considered when drawing conclusions and for future studies.

### Distribution

The distribution of respondents across the Spanish territory was variable, and some areas were not represented. Moreover, the volume of responses is not sufficient to draw local conclusions or provide a true representation of opinions at the national level. For these reasons, conclusions should be regarded as a sample from a small target population in Spain, without considering it as a true national representation.

### Participant profile

Sufficient information about who actually responds to a survey in relation to the questions asked is essential in determining whether inferences from survey research are valid (Holtom *et al*., 2022). In this study, professionals from different eye health sectors have participated in the survey, which adds to the study’s validity.

The profile of the participants, according to their job location, was very similar between those who work in a hospital and those who work in a clinic, with a minority working in universities. Nearly half of the respondents had more than 10 years of professional experience, while the other half had less than 10 years. Therefore, it can be concluded that there is a balanced representation of professionals in hospital and clinical positions, with a minority in university positions. According to the academic qualification, it can also be concluded that more than half of the respondents have qualifications higher than a bachelor’s degree.

### Interest in the inclusion and promotion of the orthoptics profession in Spain

Around 90% of respondents advocate for including orthoptists in Spanish units for strabismus, ocular motility, neuro-ophthalmology, and paediatric ophthalmology. However, this strong support, coupled with acknowledged benefits in enhancing care, might be influenced by the pre-survey informative document or Likert scale biases.

Figure 7 evaluates the possible relationship between years of experience and opinions on the necessity of incorporating orthoptists in various ophthalmology units in Spain (Q5). Despite some variations, most responses in all groups of years of experience are positive, with only a small percentage being negative. There is a slight uptick in negative responses with more experience, but due to limitations in the survey, no definitive conclusions can be drawn regarding the relationship between experience and response type.

Regarding enhancing care quality and reducing waiting lists through ophthalmologist-orthoptist collaboration, almost 90% of respondents believe it would significantly improve both areas. Additionally, 83% of participants emphasise the importance of Spanish ophthalmological societies and associations aligning with global medical organisations to advocate for orthoptists.

### Interest in requesting orthoptists, level of orthoptist training and level of involvement in their training

The majority of participants expressed support for requesting orthoptists into their work settings and emphasised the need for advanced training. Specifically, 83% of respondents showed interest in having orthoptists in their work environment, either being interested or very interested. Additionally, nearly 90% of participants emphasised the necessity for orthoptists in Spain to attain the advanced training level outlined by the EDORTH project (EDORTH, 2024; OCE, 2021).

Once again, a high ratio of answers showed interest in requesting orthoptists and agreed on the importance of advanced training for orthoptists according to European competencies. This high percentage of positive answers could be influenced by the cited flaws in designing the questionnaire and the impact of the informative report.

However, when assessing the level of involvement to determine if there are enough professionals interested in training orthoptists, it is noteworthy that a quarter of participants were neutral. Support from the medical community is essential for developing potential educational programmes. The design of university study plans facilitates acquiring official qualifications, as outlined in the Spanish Law of Health Professions, which states that ‘a health profession, titled and regulated, must have specific pre-graduate or specialised training for health care’ (LOPS, 2003). Additionally, Spanish law regarding the University System indicates that ‘the function of the University System is the preparation for the practice of professional activities’ (LOSU, 2023). Therefore, the support of the medical community in training is a fundamental aspect to be considered in further studies. This result could indicate that seeking support to train orthoptists might become a challenging task.

### Consistency of Responses

Figure 8 illustrates that the majority of response pairs are located in the upper right quadrant. These results further support the earlier conclusions regarding blocks 2 and 3. In other words, the respondents agree that the inclusion of orthoptists in Spain is necessary, along with a strong emphasis on the importance and interest in almost everything proposed in blocks 2 and 3. This includes enhancing the quality of healthcare, promoting orthoptists within Spanish medical societies and associations, ensuring orthoptists’ competencies are of an advanced standard and expressing interest in their collaboration with ophthalmologists.

### What is expected from an orthoptist?

Thanks to the open-ended questions, opinions, comments, proposals and suggestions from participants were gathered, concluding that:

- Orthoptists enhance efficiency for strabismologists and provide essential patient support by assessing binocular vision and ocular motility, treat children using eye patches, prism patients, and those needing visual re-education. They also measure versions and ductions, and deviations, and collaborate in binocular vision treatments, refraction, oculomotor control studies, and low vision care.
- Collaboration and teamwork are vital, with orthoptists working closely with ophthalmologists and other healthcare professionals, integrating seamlessly into teams and complementing patient care.
- Professionalism, patience, and initiative are crucial for high-quality, patient-centred care.
- Proper training and aligning orthoptist education with international standards are deemed essential to support patient care and meet healthcare system demands.
- Orthoptists should collaborate with other healthcare professionals rather than replacing them, clarifying their distinct skills whilst opposing medication administration.
- There is an absence of orthoptists in the Spanish National Health System, underlining the importance of collaboration with ophthalmologists, particularly for strabismus consultations and patient care in ophthalmology services.

#### Orthoptic therapy versus behavioural visual therapy

Several comments have raised concerns about non-evidence-based visual therapies, emphasising the need to distinguish these from the profession of orthoptics. To address potential aversion to supporting the introduction of orthoptists, it is essential to explain the distinctions between orthoptic therapy and behavioural vision therapy.

Therapies designed to target specific binocular dysfunctions are known as orthoptic visual exercises. This orthoptic therapy used by ophthalmologists, orthoptists, and optometrists is directed at improving visual acuity, ocular alignment, or both and relieve ocular symptoms (Rucker and Phillips, 2018). Orthoptists are trained to diagnose and manage visual defects, promote binocular vision, and correct deviations using methods like lenses, prisms, and patches. Vision therapy by orthoptists is a form of treatment to restore binocular single vision and enhance the control of a deviation thereby relieving ocular symptoms. It predominantly focuses on addressing convergence insufficiency, with consensus among reviewed studies that vision therapy is highly effective for recovering from symptomatic convergence insufficiency in both adults and children (Mohamed and Alrasheed, 2023; Rucker and Phillips, 2018; Schulman *et al*., 2024; Shainberg, 2010; Singh, 2011).

Behavioural vision therapy aims to address visual disorders, visual stress, and learning disabilities in children through approaches that differ from conventional optometric and orthoptic practices. These alternative methods often lack scientifically validated outcomes and measurable effectiveness, raising concerns about their potential harm to children (Barrett, 2009; Rucker and Phillips, 2018; Shainberg, 2010; Wang and Kuwera, 2022). Consequently, several professional organisations in Spain have emphasised the risks associated with these widely adopted practices and have publicly expressed their disapproval (SEDOP, 2019; SEOptometria, 2019).

In conclusion, it is important to emphasise that orthoptics as a profession is entirely separate from non-evidence-based visual therapy or behavioural vision therapies.

#### The role of orthoptists in the eye care team in Spain

In Spain, healthcare is a universal right, ensuring health coverage for the entire population through public funds. The National Health System serves as the primary healthcare provider for 96.5% of the population, offering comprehensive eye care services, including diagnosis, treatment, and rehabilitation by ophthalmology specialists (SNHS, 2021).

The eye care team, led by ophthalmologists, includes optometrists, nurses, opticians, medical assistants, technicians, and photographers, all working together to provide comprehensive eye care (AAO, 2024; WHO, 2022b). Proper organisation of activities and roles within the team is crucial to avoid duplication, minimise inefficiencies, and maximise resources (WHO, 2022b).

In Spain, the visual care team includes ophthalmologists, optometrists, and ophthalmic nurses, each playing distinct roles. An ophthalmologist (eye doctor) is a physician specialised in all aspects of eye health, including the diagnosis, treatment, and surgery of eye diseases and visual disorders (OftalmoSEO, 2024). Optometrists are the primary healthcare practitioners of the eye and visual system who provide comprehensive eye and vision care, which includes refraction and dispensing, detection/diagnosis and management of disease in the eye, and the rehabilitation of conditions of the visual system (WCO, 2020). Ophthalmic nurses are trained with clinical and scientific knowledge to evaluate, diagnose, treat, and discharge patients with ocular conditions and diseases/disorders (MORADI, 2016).

Orthoptics, established in collaboration with paediatric ophthalmology, aims to improve strabismus care for children (Miller, 2015). Orthoptists receive extensive training in strabismus and paediatric ophthalmology, enabling them to independently see patients of all ages under an ophthalmologist’s sponsorship (AAPOS, 2024). Based on this information, orthoptists in Spain are likely to work in units specialising in strabismus, ocular motility, neuro-ophthalmology, and paediatric ophthalmology.

## Conclusion

Given the absence of the orthoptic profession in Spain, a nationwide survey was initiated to gauge the interest of Spanish paediatric ophthalmologists and strabismologists in introducing the orthoptic profession in the country.

As participants were encouraged to review a comprehensive report on orthoptics, caution is warranted when extrapolating results from surveys due to potential bias influencing positive results. Furthermore, methodologically, it is acknowledged that the design of questions and answers deviated from typical Likert scale norms, which could lead to inaccurate conclusions.

Responses were received from 42 clinicians within the target ophthalmologists situated in various regions across Spain. The sampling approach was limited by the small target population and resource constraints. Therefore, this should be considered a sample rather than a true national representation in Spain.

From the received surveys, a typical profile emerged among participants. They predominantly operate in hospital public health services, hold advanced degrees such as master’s or doctorate degrees, and have over three years of experience in paediatric ophthalmology and strabismus clinics.

Many respondents strongly endorse integrating orthoptists into their professional environments, emphasising the need for rigorous training and competence. Furthermore, there is consensus on promoting the orthoptics profession within Spanish ophthalmological societies.

In assessing the level of involvement to gauge interest in training orthoptists, it’s notable that a quarter of participants remained neutral. This result suggests that obtaining support for orthoptist training could be challenging.

The results underscore the urgent need for recognising, supporting, and promoting the orthoptics profession within Spanish medical societies and associations. The survey also highlights critical insights, including the call for enhanced infrastructure and resources for orthoptists in hospital settings and the importance of distinguishing the orthoptics profession from behavioural visual therapies or behavioural optometry.

This survey represents an initial sample survey in Spain to gather the perspectives of paediatric ophthalmologists and strabismologists regarding the orthoptics profession. Despite methodological errors, the contextualised conclusions drawn from this study inform future research endeavours and provide valuable evidence of support towards regulating the orthoptics profession in Spain.

## Additional File

The additional file for this article can be found as follows:

10.22599/bioj.359.s1 Annex. This section includes supplementary data on responses, the Spanish version of the questionnaire, and information about the EDORTH project.
