# Supplementary material for: Enhancing Paediatric and Strabismus Ophthalmology Departments in Spain Through the Inclusion of Orthoptists: Insights from a Nationwide Survey
Source: Br Ir Orthopt J. 2024 Dec 3;20(1):241–57. doi: 10.22599/bioj.359 (PMC11623091; doi:10.22599/bioj.359)
Supplement: Annex. — This section includes supplementary data on responses, the Spanish version of the questionnaire, and information about the EDORTH project. [file bioj-20-1-359-s1.pdf]

Annex

Spanish version of the questionnaire

Cuestionario tras la lectura del artículo: CALIDAD ASISTENCIAL Y CAPACITACIÓN DE ORTOPTISTAS EN CONSULTAS DE OFTALMOLOGÍA PEDIÁTRICA Y ESTRÁBICA.

1. **Nombre de la ciudad donde trabaja.**

2. **Puesto de trabajo (puede marcar más de una opción):**
   - Hospital
   - Clínica
   - Universidad

3. **Años de experiencia profesional en consultas de estrabología y oftalmología pediátrica.**
   - Menos de 3 años
   - De 3 a 6 años
   - De 6 a 10 años
   - Más de 10 años

4. **Formación académica:**
   - Licenciado
   - Máster
   - Doctorado

5. **¿En qué grado piensa que es necesaria la inclusión de la profesión de ortóptica en unidades de estrabismo, motilidad ocular, neuroftalmología y oftalmología pediátrica en España?**
   - No es necesaria
   - Poco necesaria
   - Neutral
   - Necesaria
   - Muy necesaria

6. **¿En qué grado cree que la colaboración oftalmólogo-ortoptista mejoraría la calidad asistencial y la disminución de listas de espera**?
   a. No mejora
   b. Mejora Poco
   c. Neutral
   d. Mejora
   e. Mejora mucho

7. **¿En qué medida piensa que es importante que la sociedades y asociaciones oftalmológicas españolas se equiparen a las demandas de promoción de ortoptistas, tal y como hacen las organizaciones médicas internacionales?**
   a. No es importante

b. Poco importante
c. Neutral
d. Importante
e. Muy importante

8. **¿En qué medida cree necesario que en España las competencias de los ortoptistas cumpliesen con el más alto nivel de formación, según lo estrablecido en el proyecto EDORTH?**
    a. No es necesaria
    b. Poco necesaria
    c. Neutral
    d. Necesaria
    e. Muy necesaria

9. **¿En qué medida le interesaría demandar ortoptistas en su ámbito de trabajo?**
    a. No me interesa
    b. Me interesa poco
    c. Neutral
    d. Me interesa
    e. Me interesa mucho

10. **¿Le interesaría involucrarse en la formación de ortoptistas, si así fuera requerido?**
    a. No me interesa
    b. Me interesa poco
    c. Neutral
    d. Me interesa
    e. Me interesa mucho

11. **¿Qué esperaría de un ortoptista, si pudiese trabajar con él?**

12. A continuación, puede aportar su **opinión, comentarios**, **propuestas o sugerencias**.

Gracias por su colaboración.

Todos los resultados que pudieran ser publicados serán totalmente anónimos, lo que significa que no será posible identificar a ningún participante.

EDORTH project

EDORTH project. There are differences in the level of training of orthoptists between European countries. To harmonise training programs throughout its territory and raise them to the advanced level, the OCE (Orthoptistes de la Communauté Européenne)(OCE, 2022b) launched the EDORTH (Education Orthoptics) project (EDORTH, 2024), which was financed by Erasmus+. Thanks to this project, in 2021, the professional skills that all orthoptists must have, were established in order to be recognised in Europe. If these skills are obtained, orthoptists can apply for the European diploma by passing an exam. The

condition of access to this exam is that orthoptists have been trained by ophthalmologists and orthoptists both in theory and in practice. The acquisition of this diploma will ensure the advanced level of training for orthoptists throughout the European space. It will help also to standardise and facilitate the orthoptist´s mobility and recognition (OCE, 2024, 2021).

The competencies are outlined based on the advanced level of orthoptists´ training (OCE, 2021)

Table A.1: Levels of knowledge and competence

	**Level of knowledge**	**Level of competence**
4		**Advanced**
	• specialist knowledge<br>• specific orthoptic knowledge where other professionals might ask	• specialist skill<br>• a specific orthoptic skill where other professionals might ask the orthoptists advice
3		**Intermediate**
	• core knowledge in a straightforward situation<br>• recognition of personal limits of knowledge, needs help in more complex fields	• core skill in a straightforward situation<br>• recognition of personal limits of skill with support needed in more complex cases
2		**Limited experience**
	• some theoretical knowledge	• limited practical skill and might need help when performing this skill
1		**Fundamental awareness**
	• knowledge of basic principles	• basic practical skills

Table A.2: Knowledge

Level 4 knowledge
Refraction
Refractive errors: myopia, hypermetropia, astigmatism, anisometropia<br>Presbyopia
Visual functions
Visual acuity: Principles, Detection / resolution / recognition acuity / hyperacuities<br>Foveal vs peripheral vision
Binocular vision / correspondence
Basic principles of sensory fusion: Horopter / PANUMs area and space, Physiological diplopia, Retinal rivalry, Sensory fusion and stereopsis, Projection and normal correspondence
Physiology of ocular alignment, muscle laws (HERING, SHERRINGTON, LISTING), Vergence and motor fusion
Accommodation / convergence
Accommodation
Accommodation / convergence relationships (AC/A and CA/C relationships and ratios)
Special anatomy and physiology
Extraocular muscles: Structure and anatomy, Muscle actions
Orbital fascia including muscle pulleys
Ocular innervations: sympathetic and parasympathetic, cranial nerves II, III, IV, VI
Physical Optics
Lenses: optics, notation, transposition of prescriptions, lens types
Prism: optics and notation including Fresnel prisms, prism placement e.g. Prentice vs frontal plane / Effects of stacking
Ophthalmoscopes including fixation ophthalmoscopes
Retinoscopes
Effects of general disease on ocular motility and sensory function
Multiple sclerosis
Myasthenia gravis
Thyroid eye disease
Awareness of medicines used in ophthalmology
Pharmacokinetics of miotic, mydriatic, local anaesthetic and cycloplegic drugs

Table A.3: Assessment

Level 4 competences
**History taking**
Relevant orthoptic, general ophthalmic and medical history<br>Social, family, drug history
**Assessing visual functions**
Vision tests in infants and non-verbal patients (Preferential looking and vanishing optotypes, picture tests e.g. Kays, LEA symbols, tumbling E, Letter tests for children)
Vision tests in adults / verbal patients (Landolt C, letters, numbers and text reading tests)
Assessment of crowding / separation difficulty
Assessment of eccentric fixation
Confrontation fields
**Assessing ocular alignment**
Assessment of corneal reflections, knowledge of angle kappa (alpha/lambda)
Cover/ uncover test / alternate cover test
Prism cover test in primary position and 9 positions of gaze<br>Alternating, unilateral, simultaneous prism cover test
Synoptophore: horizontal/vertical /torsional angle of deviation, objective and subjective angle,…
Methods using diplopic projection (Maddox Rod, von Graefe, scale methods)
**Assessing ocular motor functions**
Ocular motility: versions and ductions, smooth pursuit and saccades, vergence, translatory movements, vestibulo-ocular reflex (VOR), optokinetic nystagmus (OKN)
Lees screen / Hess chart / Harms tangent screen
Field of uniocular fixation
Field of binocular single vision
Convergence to near point
Clinical assessment of nystagmus
**Assessing binocular functions / correspondence**
Prism fusion: ranges (to blur, diplopia and recovery), 4 prism test, 20 Base out prism test
Relative fusion/relative vergence methods
Bagolini striated glasses
Worth's lights
Synoptophore and prism assessment of potential binocular function

Table A.4: Diagnosis

Level 4 competences
Visual functions
Amblyopia: strabismic, anisometropic, combined mechanism, ametropic, stimulus deprivation
Eccentric fixation
Ocular alignment
Excluding / unveiling pseudo strabismus
Large or decompensating esophorias
Intermittent esotropia: fully accommodative, convergence excess
Distance esotropia in the elderly
Cyclic strabismus
Constant esotropia: infantile, constant without abnormal correspondence (AC), constant with AC, with accommodative element (partially accommodative), microtropia
Large or decompensating exophorias
Intermittent exotropia: distance and non-specific exotropia, near exotropia
Consecutive strabismus
Secondary strabismus
Vertical deviations in primary concomitant strabismus e.g. inferior oblique dysfunction
Dissociated vertical or horizontal divergence (DVD /DHD)
Accommodation and convergence disorders
Convergence insufficiency, paralysis
Convergence spasm
Accommodation inertia / insufficiency / paralysis
Accommodation spasm
Ocular motility disorders / incomitant strabismus
IIIrd nerve palsies
IVth nerve palsies
VIth nerve palsies
Differential diagnoses (recent/longstanding, neurogenic/myogenic)
Alphabet patterns (A/V/Y/X/λ)
Orbital trauma and fractures
Incomitant strabismus associated with high myopia ("heavy eye") or healthy ageing (distance esophoria/"sagging eye syndrome")
Browns syndrome
Duanes syndrome
Other congenital cranial dysinnervation syndromes (CCDDs) including "congenital fibrosis syndrome" / Marcus Gunn syndrome
Ptosis
Obliquus superior myokymia
Nystagmus
Idiopathic and infancy onset nystagmus: Orthoptic management, surgical management
Latent nystagmus

of the European Union

Table A.5: Therapy

Level 4 competences
Optical management
Principles of spectacle prescription in non-strabismic children and in strabismic children and adults, especially where the prescription affects the angle of deviation
Prism prescription / incorporated prisms for children and adults / press-on prism fitting
Amblyopia therapy
Occlusion (patches)
Optical penalisation
Atropine penalisation
Cycloplegia in accommodative problems
Orthoptic exercises
Convergence /divergence methods (prisms, synoptophore, dot card etc.)<br>Relative vergence/accommodation methods (positive /negative relative vergence/accommodation, stereograms)

Supplementary Data

Table A.6: Number of responses from Block 2.

Q5			Q6			Q7		
**Answers**	**Number**	**%**	**Answers**	**Number**	**%**	**Answers**	**Number**	**%**
**Very necesssary**	23	54.8	**Significantly improves**	21	50.0	**Very important**	16	38.1
**Necessary**	14	33.3	**Improves**	16	38.1	**Important**	19	45.2
**Neutral**	3	7.1	**Neutral**	3	7.1	**Neutral**	4	9.5
**Slightly necessary**	1	2.4	**Slightly improves**	1	2.4	**Slightly improtant**	3	7.1
**Not necessary**	1	2.4	**Does not improves**	1	2.4	**Not important**	0	0.0
**Total**	42		**Total**	42		**Total**	42	
